# Effects of human blood red cells on the haemolytic capability of clinical isolates of *Candida tropicalis*

**DOI:** 10.1186/s12929-015-0120-8

**Published:** 2015-02-11

**Authors:** Marcia Cristina Furlaneto, Daniel Favero, Emanuele Julio Galvão França, Luciana Furlaneto-Maia

**Affiliations:** Department of Microbiology, Paraná State University at Londrina, C.P. 6001, 86051990 Londrina, Brazil; Technological Federal University of Paraná, Paraná, Brazil

**Keywords:** Haemolytic activity, Putative *HLPt* gene, Erythrocytes

## Abstract

**Background:**

*Candida tropicalis* is an increasingly important human pathogen associated with high mortality rates; however, little is known regarding the virulence properties of *C. tropicalis*, particularly the production of haemolytic factor. Although *Candida* spp may acquire iron from human blood red cells (RBCs) by producing a haemolytic factor that promotes cell lyses, at present there are no data regarding the effect of RBCs on the production of haemolytic molecules. The present study was undertaken to evaluate the role of human red blood cells on the production haemolytic factor by *C. tropicalis*; in addition, the transcription levels of a putative haemolysin-like protein gene (*HLPt*) were also analysed.

**Results:**

*C. tropicalis* isolates produced a haemolytic factor following growth in either the absence or presence of RBCs; however, distinct levels of haemolysis were observed, with 60% of the isolates exhibiting a significant increase in the production of haemolytic factor when grown in the presence of human RBCs. All isolates in which the putative *HLPt* gene was up-regulated in presence of human RBCs, ranging from 1.044 to 6.965-fold, also exhibited higher haemolytic activity following growth in the presence of RBCs compared to that observed in the absence of RBCs.

**Conclusions:**

We propose that human RBCs may induce changes in the phenotypic expression of haemolytic factor and in transcriptional levels of the putative *C. tropicalis HLPt* gene in an isolate-dependent fashion.

## Background

Non-*Candida albicans Candida* (NCAC) species are being increasingly reported as causative agents of severe systemic infections [1,2]. Among NCAC species, *Candida tropicalis* is one of the most commonly isolated species in tropical countries. For instance, according to recent epidemiological surveys, *C. tropicalis* is one of the main causal agents of candidemia in Latin America and Asia [3-7].

*C. tropicalis* is taxonomically close to *Candida albicans*, sharing several pathogenic traits [8]. The clinical importance of *C. tropicalis* infections is associated with high rates of morbidity and mortality that may exceed those reported for other *Candida* species, including *C. albicans* [9,10]. However, compared with *C. albicans,* there are relatively few studies examining the virulence attributes of *C. tropicalis*.

Haemolytic activity is known to be a putative virulence factor contributing to disseminated candidal pathogenesis, particularly facilitating hyphal invasion in disseminated candidiasis [11,12]. The haemolytic factor of *C. albicans* is a mannoprotein, which has a cell-wall mannan sugar moiety structure [13]. The mannan fraction binds to the erythrocyte membrane, causing its rupture and allowing the release of haemoglobin which may be used as an iron source by the yeast [13-15].

The haemolytic capability of *C. tropicalis* has also been recognized as a putative trait related to its pathogenesis [16-20]. We have previously reported that a haemolytic factor produced by clinical isolates of *C. tropicalis* is released in the culture supernatant and that its production occurs in a glucose-dependent manner [19]. More recently, our group demonstrated that binding of the secreted haemolytic factor to lectin Concanavalin-A reduced the haemolytic activity of *C. tropicalis* to similar level as that observed for *C. albicans*, suggesting that the haemolytic factor secreted by *C. tropicalis* may be a mannoprotein and giving evidence for its involvement in haemolysis [20].

Currently, there are still few data concerning the molecular basis of haemolytic factor production by *Candida* species. Lachke et al. [21] have described a putative haemolysin gene in *Candida glabrata* termed *HLP* (haemolysin-like protein). Further, Luo et al. [22] demonstrated a positive correlation between the expression of *HLP* and haemolytic activity in *C. glabrata*.

In this study, we demonstrated for the first time that human red blood cells modulate the production of the haemolytic factor in *C. tropicalis*; furthermore, our data revealed a positive correlation between the mRNA expression of a putative haemolysin-like protein gene and the haemolytic activity in *C. tropicalis* isolates, which suggests a positive effect of RBCs on haemolysis.

## Methods

### *Candida tropicalis* isolates

Clinical isolates recovered from blood (136.06, 144.06, 189.06, 301.07), nail infections (151.06) and tracheal secretions (197.06, 201.06, 254.07, 344.07, 335.07) [23] were maintained as stock cultures from the Fungal Genetics Laboratory, The University of Londrina-Brazil on yeast extract-peptone–D-glucose (YPD) agar at −20°C in glycerol.

### Preparation of red blood cells (RBCs)

Human RBCs (A^+^ type) were centrifuged at 1080 g for 7 min. The supernatant and buffy coats were removed, and the packed RBCs were resuspended in Ca^2+^- and Mg^2+^ -free phosphate-buffered saline (PBS-) and washed twice with the same PBS-buffer and centrifugation. The RBCs were then added to liquid RPMI 1640 medium without phenol red (Sigma Chemical Co., St. Louis, MO, USA) at a final concentration of 10^8^ cells/ml.

### Growth conditions

Prior to cultivation in either the presence or absence of human RBCs, *C. tropicalis* isolates were grown overnight to stationary phase in RPMI at 37°C and 180 rpm. The procedure was repeated, after which cells were harvested by centrifugation (3000 g for 10 min) and washed with sterile buffer (PBS-). The resultant suspensions were then inoculated with 10^4^ yeast/ml in RPMI medium and in RPMI supplemented with human RBCs type A^+^ (10^8^ cells/ml) followed by incubation at 37°C and 180 rpm for 48 h. Finally, cells were harvested by centrifugation (3000 g for 5 min) and used for RNA extraction. The resulting culture supernatants were assayed for haemolytic activity.

### Haemolytic activity in the cell-free broth

Haemolytic activity was determined as described previously [19]. Briefly, the culture supernatants were filtered (0.22 μm membranes, Millipore MILLEX GV) to ensure the complete cell removal and concentrated 2.5-times by freeze-drying followed by storage at −20°C. The cell-free culture supernatant and red blood cells (RBCs) suspended in RPMI medium (1 × 10^8^ cells/ml) were mixed at a 1:1 (v/v) ratio, and incubated at 37°C for 15 h. After the incubation, samples were centrifuged at 1000 g for 2 min. Absorbance of the supernatant was determined at 405 nm. Haemolysis was calculated according to the equation: Haemolysis (%) = 100 ‐ [(Ap ‐ As)/(Ap ‐ An) × 100)]; where Ap, As and An are the absorbance of the positive control, test sample and negative control, respectively. The positive control was the RBCs lysed with SDS (0.6%) plus PBS- buffer at a 1:1 (v/v) ratio, and the negative control was the human red blood cell suspension with RPMI plus PBS- buffer at a 1:1 (v/v) ratio. The results are given as the ratio between the haemolysis percentage of the test condition and control. The experiments were performed three times.

### A putative *C. tropicalis* haemolytic factor encoding gene

A putative *C. tropicalis* orthologue gene to *HLP*, which encodes a haemolysin-like protein gene in *C. glabrata*, was searched using *HLP* specific primers [22] against *C. tropicalis* genomic DNA obtained as described previously [24]. The amplicon of a *C. tropicalis* putative *HLPt* (haemolysin-like protein) gene was generated in 50 μl of a reaction mixture containing 4 μl DNA (5 ng/μl), 612.5 μmol of each dNTP (Invitrogen, Carlsbad, CA, USA), 40 pmol of each primer, and 2.5 U of Taq polymerase (Invitrogen, Carlsbad, CA, USA). PCR reactions were performed at 94°C for 5 min, followed by 35 cycles of 30 s at 94°C, 30 s at 56.6°C and 1 min at 72°C, with an additional extension (72°C, 10 min). All the PCR reactions were performed in a GeneAmp PCR system (Eppendorf, Mastercycler gradient). The amplicon obtained was sequenced using the DYEnamic ET dye Terminator Cycle Sequencing Kit (Amersham Pharmacia Biotech, Inc) on MegaBACE 1000. Characteristics of the obtained sequence such as homologies and the hydrophobicity profile were predicted by online tools. The alignment and comparison of the nucleotide and aminoacid sequences with sequences in the databases were performed using BLAST tools available on the NCBI (htpp://www.ncbi.nlm.nih.gov) and UniProt (http://www.uniprot.org) websites. Transmembrane region prediction was performed using TMpred (http://www.ch.embnet.org/software/TMPRED_form.html).

### Reverse transcription–qPCR (RT-qPCR)

*HLPt* primers were designed for RT-qPCR analysis using Gene Runner 3.05 software (http://www.generunner.com). β-actina (*ACT*) was used as a reference housekeeping gene (Table 1). Primers had melting temperatures (Tm) between 58°C and 60°C, and amplification products were 129 and 151 bp, respectively. The oligonucleotides did not show, when analysed by themselves or together, hairpin loops, dimmers, bulge loops or internal loops according to program analyses. Primer specificity was checked using BLAST analysis (*Basic Local Alignment Search Tool*, http://blast.ncbi.nlm.nih.gov) searches against the GenBank database. Standard gel electrophoresis using a 2% TBE gel was used to check for a single product of the expected size.

**Table 1:** **Primer sequences employed in the analyses of** ***HLPt*** **-mRNA expression and respective amplicon sizes**

**Gene**	**Orientation**	**Sequence (5′ → 3′)**	**Amplicon size**	**Reference**
HLPt*	FW	TAGTGGGGCAAGTAGTGGG	151 pb	This study
	RV	GGTGGTGATAGATGTAGCAG		
Β-Actin	FW	AACCTCTTCTCAATCATCTGC	129 pb	Vandeputte et al. [25]
	RV	GCTTCCAAACCTAAATCGGC		

*HLPt primers were designed based on the complete ORF of the putative *C. tropicalis* haemolysin-like protein gene sequence using Gene Runner 3.05 software.

The assessment of differential expressions of the *HLPt* gene by *C. tropicalis* isolates was performed following growth in the absence of RBCs (RPMI medium) (control) and in RPMI supplemented with RBCs (test condition). After 48 h of culture, *C. tropicalis* cells were harvested, washed using distilled sterile water, suspended in Trizol® (Invitrogen, Carlsbad, CA, USA) and frozen in liquid nitrogen. Total RNA was extracted with Trizol®, according to the manufacturer’s instruction and suspended in 30 μl of DEPC-treated water. Residual genomic DNA was eliminated by DNAse I (Invitrogen, Carlsbad, CA, USA). Purity of the extracted RNA was determined by the 260/280 nm ratio, and integrity was checked by electrophoresing on 1% agarose gel. RNA was stored at −80°C until further use and quantified with a Nanodrop ND-1000 Spectrophotometer (NanoDrop Technologies Inc.). Reverse transcription was carried out using 500 ng total RNA previously incubated at 64°C for 10 min and snap-cooled on ice for 2 min to denature. First-strand cDNA was synthesized by the addition of MgCl_2_ (4 mM), 2 μl of 10x PCR Buffer, dNTP mixture (200 μM), Oligo (dT)-18 primer (0.4 μM), RNase OUT (4 U) and reverse transcriptase RT M-ML V (0.5 U) (Invitrogen, Carlsbad, CA, USA). The mixture was incubated at 42°C for 60 min. Synthesis reactions were performed in a GeneAmp® PCR system (Eppendorf, Mastercycler gradient).

Real time PCR was carried out in a PTC-200 DNA Engine Cycler (MJ Research) with a Chromo4™ Four-Color Real-Time System (Bio-Rad). The 20 μl reaction was performed by using the Platinum® SYBR® Green qPCR Supermix-UDG (Invitrogen, Carlsbad, CA, USA), 20 μM of each primer, and 6 μl of a 1:20 cDNA dilution of each isolate in their respective growing conditions. The negative controls (with no DNA template) for each primer set were included in each run. Real-time PCR reactions were performed at 50°C for 2 min, 95°C for 5 min, followed by 40 cycles of 30 s at 94°C, 30 s at 56,6°C and 1 min at 72°C, with an additional extension (72°C, 10 min). Melting curve analysis was performed for each sample to assure that a single product was produced in each reaction.

RT-qPCR data were normalized with the constitutively expressed β-Actin gene (*ACT*) [25]. Normalized data were then used to calculate the relative gene expression levels. An expression level corresponds to the expression of the *HLPt* gene in the presence of RBCs relative to its expression in the absence of RBCs.

Data from RT-qPCR reactions were previously analysed using MJ Opticon Monitor TM Analysis Software (MJ Research) for determination of C_q_ (Quantification cycle) for each reaction and their respective efficiencies of amplification. The C_q_ value for the *HLPt* and *ACT* genes was measured, and the expression level of *HLP* for the different isolates was analysed using the efficiency adjusted normalization method calculated by the formula: Ratio = (E _target_)^ΔCq target(Mean control-Mean sample)^/(E _ref_)^ΔCq ref(Mean control-Mean sample)^ [26], representing the x-fold difference from the calibrator (*ACT*). To determine the changes in the relative gene transcription level presented as fold changes, a mathematical model for relative quantification was used [27]. Three independent experiments (biological and technical replicates) were performed in each condition, including growth, RNA extraction and qRT-PCR.

### Statistical analysis

SPSS 17.0 software (SPSS Inc.) was used in the statistical analyses of haemolytic activity. A Kruskal-Wallis test was used to evaluate the significant differences in haemolytic activity promoted by the isolates grown in each of the two growth conditions. The relative expression software tool REST© [26] was used to determine the relative expression of the *HLPt* gene in the different culture conditions evaluated and to test whether the expression differences were significant. Gene expression in the test condition was considered significantly different, either by induction or repression, from the control when p < 0.001.

## Results

### Effect of red blood cells on production of haemolytic factor

**Figure 1:**
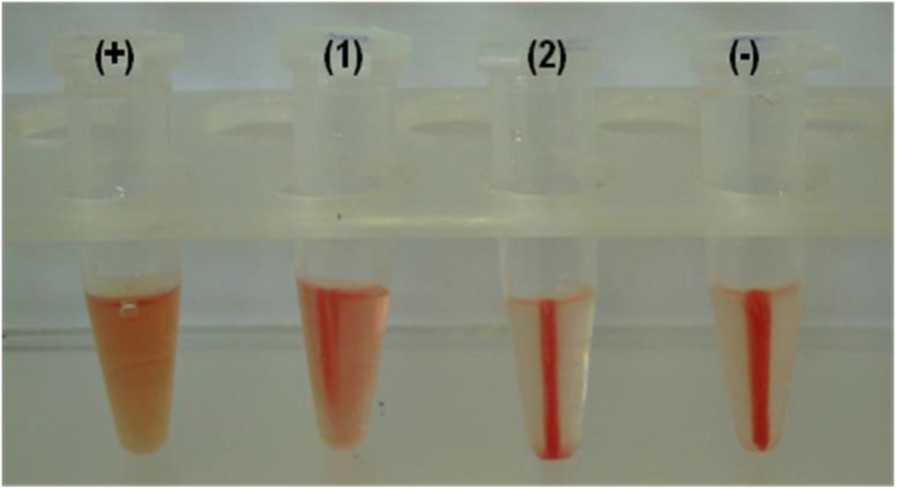
**Representative photograph showing haemolysis promoted by the haemolytic factor produced by** ***C. tropicalis*** **.** For haemolysis assays, cell-free culture supernatants and RBCs were mixed at a 1:1 (v/v) ratio following incubation at 37°C for 15 h. (+) positive control (total lyses of RBCs in the presence of SDS), (1–2) haemolysis promoted by isolate 344.07 following growth in the presence and absence of RBCs, respectively, and (−) negative control (RBCs cells suspension with RPMI plus PBS buffer).

Differences in the levels of haemolysis obtained from cell-free broth following growth in the absence and presence of human RBCs are illustrated in Figure 1. All isolates tested produced a haemolytic factor that promoted the release of haemoglobin from human RBCs. The relative haemolysis data, giving by the ratio of the haemolysis percentage obtained in cultures in the presence of human RBCs relative to that in the absence of RBCs (RPMI cultures), are shown in Table 2. For 60% of the isolates, human RBCs had a positive effect on haemolysis promotion (p < 0.05) (Table 2). Among these isolates, for isolate 189.06 (lowest relative haemolysis), the percentages of haemolysis from cell-free culture supernatants obtained following growth in the presence of RBCs relative to haemolysis in the absence of RBCs were 2.73 ± 0.1/2.57 ± 0.06; for isolate 254.07 (highest relative haemolysis), the percentages of haemolysis from cell-free culture supernatants obtained following growth in the presence of RBCs relative to haemolysis in the absence of RBCs were 3.83 ± 0.31/1.71 ± 0.34.

**Table 2:** **Effect of human red blood cells (RBCs) on haemolysis promotion by clinical isolates of** ***Candida tropicalis***

**Isolates**	**Relative haemolysis** ^**1**^	**Effect of RBCs on haemolysis promotion**
136.06	1.25	Positive*
144.06	2.12	Positive*
189.06	1.06	Positive*
151.06	1.88	Positive*
254.07	2.24	Positive*
344.07	1.67	Positive*
301.07	0.94	None
197.06	0.94	None
201.06	0.95	None
335.07	0.95	None

^1^Relative haemolysis given by the ratio of haemolysis measured from cell-free culture supernatant obtained following growth in the presence of RBCs relative to haemolysis measured from culture supernatant obtained following growth in the absence of RBCs; *positive effect significant at p < 0.05 by the Kruskal-Wallis Test.

For 40% of the isolates, no significant differences were observed in the haemolytic activity between cultures grown in the presence or absence of RBCs (Table 2). Among these isolates, for isolate 197.06 (lowest relative haemolysis), the percentages of haemolysis from cell-free culture supernatants obtained following growth in the presence of RBCs relative to haemolysis in the absence of RBCs were 1.63 ± 0.2/1.74 ± 0.08; for isolate 335.07 (highest relative haemolysis), the percentages of haemolysis from cell-free culture supernatants obtained following growth in the presence of RBCs relative to haemolysis in the absence of RBCs were 2.11 ± 0.28/2.21 ± 0.06. Additionally, to verify that RPMI medium did not promote human RBC haemolysis, a 2.5-times concentrated RPMI medium was tested by a haemolysis assay. No RBC lysis was observed (data not shown).

### A putative *C. tropicalis* haemolysin-like protein gene (*HLPt*)

**Figure 2:**
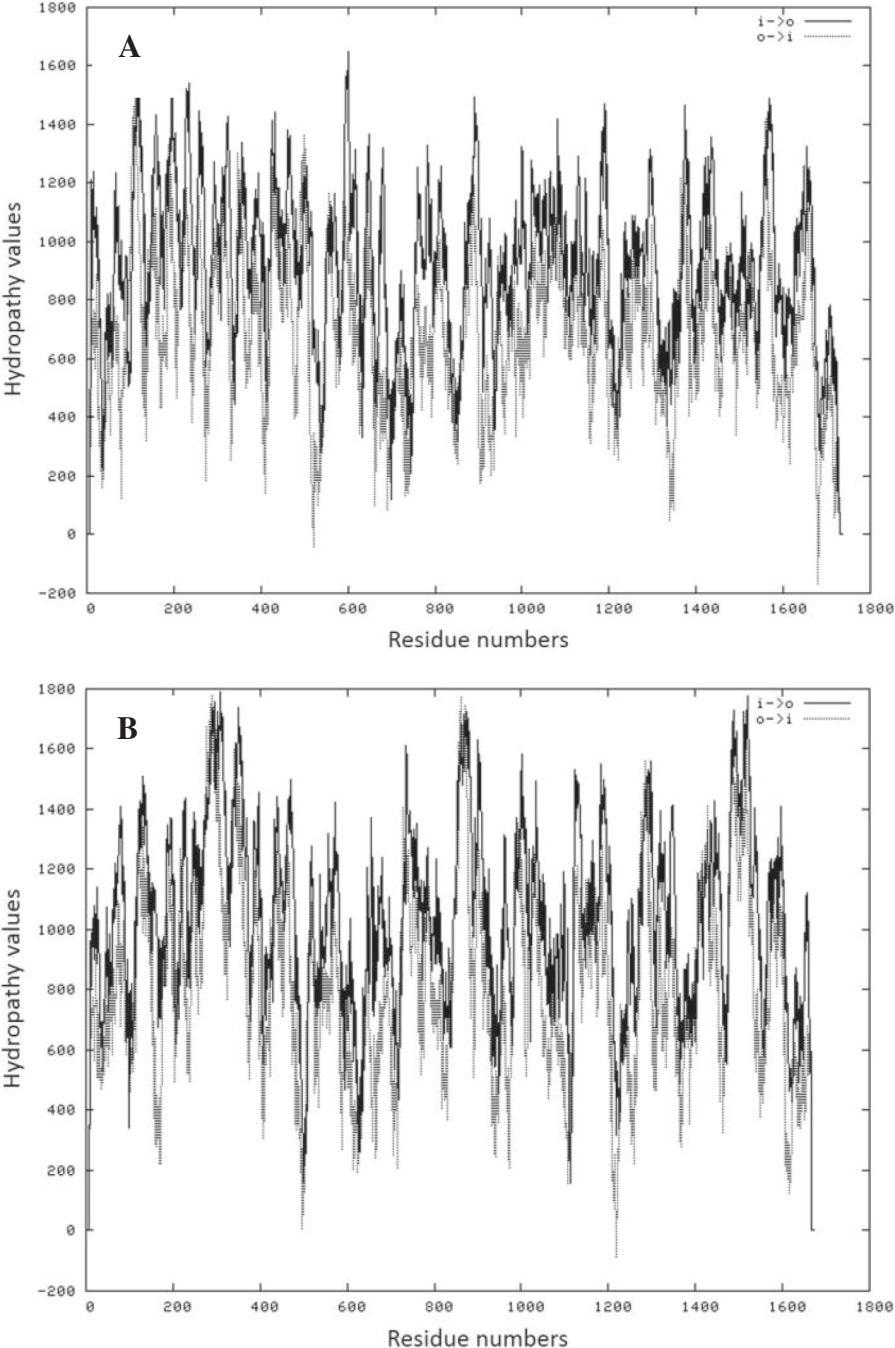
**Hydrophobicity profile obtained by TMpred analyses. (A)** TMpred obtained for *C. tropicalis* predicted protein (*Candida tropicalis* MYA-3404, GenBank: XM_002547122). **(B)** TMpred obtained for *C. albicans* hypothetical protein (CaO19.11047, GenBank: XM_709892) similar to a *Saccharomyces cerevisiae* cell wall mannoprotein (see Results section).

The employment of *C. glabrata HLP* gene primers against *C. tropicalis* genomic DNA resulted in an amplicon of approximately 400 bp (data not shown). The BLASTN analysis (BLASTN algorithm against the GenBank database) revealed that the sequence of the amplicon exhibited high identity (98%) to a *C. tropicalis* predicted protein mRNA (*Candida tropicalis* MYA-3404, GenBank: XM_002547122). The corresponding amino acid sequence of the obtained ORF had significant similarities to an uncharacterized threonine-rich GPI-anchored glycoprotein of the fission yeast *Schizosaccharomyces pombe* (EMBL Uniprot database: Q96WV6), indicating the presence of a possible domain that leads to a cell surface subcellular location. Furthermore, the *in silico* analysis inferred gene ontology of the *HLPt* gene product as a cellular component located on the external side of the cell wall. Moreover, the BLASTN analysis also revealed a high identity (70%) to a *C. albicans* hypothetical protein SC5314 (CaO19.11047, GenBank: XM_709892), annotated as an ORF similar to the DAN4p cell wall mannoprotein of *Saccharomyces cerevisiae* (EMBL Uniprot database: P47179). Hydropathy plots show comparable peak profiles in the hydrophobicity index between the predicted protein of *C. tropicalis* and the *C. albicans* hypothetical protein (Figure 2), supporting the hypothesis that the *C. tropicalis* predicted protein may be correlated to a cell wall mannoprotein, though the achieved data do not allow its classification.

The complete ORF of the *C. tropicalis* sequence was used to design gene-specific primers for a *C. tropicalis* putative haemolysin-like protein (*HLPt*) gene. The employment of these primers was successfully applied against *C. tropicalis* genomic DNA which resulted in an amplicon of 151 bp (data not shown) (Table 1).

### Expression levels of a putative haemolysin-like protein gene

Basic Local Alignment Search Tool (BLAST) analysis indicated that each primer pair employed in qPCR assays was specific for *C. tropicalis HLPt* and *ACT* genes and would not cross-react with sequences from other organisms (data not shown). Gel electrophoresis and melting curve analyses confirmed the presence of the expected qPCR products, and the absence of unwanted non-specific products, confirming that each primer pair was specific for its corresponding *C. tropicalis* gene. Non-inoculated media failed to show evidence of gene expression (data not shown). We found that the *HLPt* and *ACT* genes were expressed either in the presence or absence of RBCs at 48 h.

Table 3 shows the relative expression of the *HLPt* gene in *C. tropicalis*. The mRNA levels of the *HLPt* gene were higher in culture medium supplemented with human RBCs than in RPMI medium alone for 60% of *C. tropicalis* isolates at statistically significant (p < 0.001) levels, with an induction factor ranging from 1.044 to 6.965-fold. All isolates in which the *HLPt* gene was up-regulated in the presence of human RBCs also exhibited higher haemolytic activity following growth in the presence of RBCs compared to that observed in the absence of RBCs (Table 2).

**Table 3:** **Relative expression of the** ***HLPt*** **gene by** ***Candida tropicalis*** **clinical isolates following growth in either the absence (RPMI medium) or the presence of human red blood cells (RBCs) (RPMI + RBCs)**

	**Relative expression**	
**Isolates**	**Fold changes in mRNA expression (RPMI + RBCs ** ***vs*** **RPMI)**	**Regulation**
136.06	1.244	Up*
144.06	1.654	Up*
189.06	1.044	Up*
151.06	6.965	Up*
254.07	2.032	Up*
344.07	6.905	Up*
301.07	4.429	Down*
197.06	1.902	Down*
201.06	3.82	Down*
335.07	1.338	Down*

*Significantly different by REST Software (p < 0.001).

On the other hand, transcript levels were low for 40% of the isolates at statistically significant (p < 0.001) levels with a repression factor of 1.338 to 4.429-fold. These isolates exhibited the same extent of haemolytic activity in either the presence or absence of RBCs in culture medium.

## Discussion

Despite the clinical importance of *C. tropicalis*, studies examining virulence attributes expressed by this species are scarce. In particular, studies regarding the production of molecules exhibiting haemolytic activity by *Candida* species are limited.

The ability of pathogenic microorganisms to acquire elemental iron has been shown to be essential to their survival within the mammalian host [28]. *C. albicans* may acquire iron from human RBCs by producing a haemolytic factor that consist of a cell wall mannoprotein with its sugar fraction identified as a cell-wall mannan; the mannan binds to RBC band 3 protein, causing RBC rupture and consequent release of haemoglobin, a source of iron for the yeast [13,14].

Luo et al. [16] were the first to describe that *C. tropicalis* exhibits a haemolytic activity when grown on blood agar. We have first demonstrated that haemolytic factor is released in the culture supernatant of *C. tropicalis* isolates [19], and that it is produced by different isolates independent of their clinical origin [19,29]. Further, we demonstrated different abilities of haemolytic factor production among *Candida* species associated with bloodstream infections, where *C. tropicalis* exhibited higher haemolysis than *C. albicans* [20].

Concerning the cultures conditions under which *C. tropicalis* exhibits a haemolytic capability, it has been demonstrated that the production of haemolytic factor by this species occurs in a glucose-dependent manner [19,30], and that it is not influenced by an increased atmosphere of carbon dioxide [19], differing from that observed in the prokaryote model.

Here, we evaluated for the first time the influence of human RBCs on the production of haemolytic factor by clinical isolates of *C. tropicalis*. In agreement with previous results [19,20], the production of haemolytic molecules, following growth in RPMI medium, was a common trait of *C. tropicalis* isolates, e.g., all tested isolates produced haemolytic factor under this culture condition; however, growth in the presence of human RBCs resulted in higher haemolytic factor production for 6 out of 10 isolates tested compared to that obtained in medium where RBCs were absent. Our data revealed that human red blood cells seem to modulate the production of haemolytic factor and that it occurs in an isolate-dependent fashion. Although the haemolytic molecule produced by *C. tropicalis* has not yet been characterised at a biochemical level, we have recently suggested that it may be a mannoprotein, based on its affinity to Concanavalin A, similarly to that described for *C. albicans* [20]*.* Recently, it has been shown that *C. albicans* increases cell wall mannoproteins in response to blood [31]. According to these authors, the cell wall structure and composition, e.g., mannan chain length and complexity, are altered when *C. albicans* cells are grown in the presence of blood rather than standard laboratory growth media [31]. Moreover, it has been proposed that *C. albicans* upregulates cell wall mannan and/or mannoprotein as a defense mechanism [32]. Our data raise the hypothesis that in *C. tropicalis*, the cell wall composition and/or architecture may be altered in response to the presence of red blood cells, in a similar fashion to that observed for *C. albicans*. Currently, the influence of growth conditions on the *Candida* spp cell wall remains largely unexplored, although the cell wall is a key effector of cell morphology and an active modulator of host immune defences.

In this study, we aimed to identify a putative gene encoding a haemolysin-like protein in *C. tropicalis* that was named *HLPt* in analogy to its *C. glabrata* orthologue (*HLP*) [22]. To evaluate the transcription levels of *HLPt* we employed the quantitative real-time RT-PCR method. Our results revealed the presence of *HLPt* transcripts in all *C. tropicalis* isolates under both culture conditions tested, e.g., RPMI medium alone and RPMI supplemented with human red blood cells, suggesting that in *C. tropicalis* the *HLPt* gene may have an ubiquitous expression and that the *HLPt* gene product is a common characteristic in this species. To verify whether human RBCs modulate *HLPt* gene expression in *C. tropicalis* and to evaluate a possible correlation between the transcript levels and the profile of haemolysis promoted by different clinical isolates, the fold expression (expression level) of the *HLPt* gene was compared between the two culture conditions, e.g., absence and presence of RBCs. For the majority of isolates, the presence of RBCs upregulated *HLPt* transcription. For these isolates, we observed a positive correlation between *HLPt* induction and the production of haemolytic factor, revealed by the levels of haemolysis. On the other hand, some isolates showed RBC-repressed control of *HLPt*, although the observed haemolysis remained constant (Table 3). The reason for this absence of correlation between reduced expression of *HLPt*-mRNA and a reduction in haemolysis is unknown. One possible explanation may be related to cell density effects that may differed among isolates. Another possibility is that differences among isolates in the ability to promote the yeast-to-hypha transition may affect haemolysis levels. In another set of experiments carried out in our laboratory, we observed that *C. tropicalis* cells undertake a yeast-to-filamentous growth transition (hypha + pseudohypha) when cultured in the presence of RBCs and that this frequency is variable among isolates (unpublished data). According to Manns et al. [14], *C. albicans* hyphal cells were associated with higher haemolysis levels than blastoconidial cells.

As far as we know, this is the first study to analyse the differential expression of a putative haemolytic factor encoding gene in pathogenic *Candida* species. Future work must focus on the construction of *C. tropicalis HLPt* null mutants to evaluate environmental conditions that have effects on haemolysis.

## Conclusions

We demonstrated that human red blood cells influence the production of haemolytic factor by clinical isolates of *C. tropicalis*. In addition, this study provides evidence that RBCs induce changes in the transcription levels of the putative *C. tropicalis HLPt* gene in an isolate-dependent manner.
